# Ticagrelor-Induced Bradyarrhythmias in Acute Coronary Syndrome: A Case Series of Three Patients

**DOI:** 10.7759/cureus.116906

**Published:** 2026-09-25

**Authors:** Ashish Molleti, Ramesh Sankaran, Balakrishnan Vinod Kumar, Judah N Arul

**Affiliations:** 1 Department of Cardiology, Sri Ramachandra Institute of Higher Education and Research, Chennai, IND

**Keywords:** acute coronary syndrome, adenosine, adverse effect, bradyarrhythmia, permanent pacemaker implantation (ppm), prasugrel, ticagrelor

## Abstract

Ticagrelor is a reversible oral P2Y12 receptor inhibitor recommended in preference to clopidogrel for most patients with acute coronary syndrome. In addition to inhibiting platelets, it raises extracellular adenosine, which is thought to explain its underappreciated capacity to cause bradyarrhythmia. We report three consecutive patients with acute coronary syndrome who developed serious bradyarrhythmias after ticagrelor administration. The first, a 58-year-old woman treated with a drug-eluting stent to the left anterior descending artery, developed high-grade atrioventricular block with asystolic pauses that required a temporary pacing wire, after which ticagrelor was stopped, prasugrel was substituted, and she recovered. The second, a 48-year-old woman with an anterior myocardial infarction, had a cardiac arrest with complete heart block after ticagrelor loading, was resuscitated, paced temporarily, switched to prasugrel, and recovered. The third, a 77-year-old man with an inferoposterior and right ventricular myocardial infarction, had recurrent asystole and ventricular arrhythmia that persisted despite switching to prasugrel and required a permanent pacemaker. None of the patients were receiving an atrioventricular nodal blocking drug, and in every patient the treated coronary lesion was patent on a repeat angiogram, which made ongoing ischemia from stent failure an unlikely sole explanation. A formal Naranjo assessment rated the association as possible in each case. A ticagrelor-induced adverse effect should be considered in any patient who develops unexplained bradycardia or syncope after its administration. Early rhythm monitoring and prompt withdrawal are the key steps, and although the rhythm usually recovers, permanent pacing may be needed when conduction disease is superimposed on ischemic injury to the conduction system.

## Introduction

Ticagrelor is a reversible, direct-acting oral P2Y12 receptor inhibitor that produces faster and more consistent platelet inhibition than clopidogrel. In the PLATO (PLATelet inhibition and patient Outcomes) trial, vascular deaths, myocardial infarctions, and strokes were lower at 12 months in the ticagrelor group (9.8%) compared to the clopidogrel group (11.7%) (hazard ratio 0.84; 95%CI 0.77-0.92, p<0.001) [1].

Contemporary reviews of oral antiplatelet therapy in acute coronary syndrome (ACS) emphasize that the choice and intensity of P2Y12 inhibition must be individualized and that these agents carry specific drug-related adverse effects that clinicians must recognize [2]. Current guidance recommends a potent P2Y12 inhibitor, either ticagrelor or prasugrel, in preference to clopidogrel for most patients with ACS who undergo percutaneous coronary intervention (PCI), with clopidogrel reserved for patients in whom a potent inhibitor is unsuitable or who have an indication for oral anticoagulation [3].

Ticagrelor also has effects that are independent of platelet inhibition. In addition to reversibly antagonizing the P2Y12 receptor, it acts as an inverse agonist at that receptor and inhibits the equilibrative nucleoside transporter 1 (ENT1) [4-6]. By blocking ENT1, it reduces the cellular re-uptake of adenosine and raises the extracellular adenosine concentration, and adenosine exerts powerful negative chronotropic and dromotropic effects that slow the sinoatrial node and depress atrioventricular conduction through A1 receptors, which is thought to account for the characteristic adverse effects of dyspnea and bradyarrhythmia [4]. In the continuous electrocardiographic substudy of the PLATO trial, ventricular pauses of at least three seconds were more frequent with ticagrelor than with clopidogrel during the first week of treatment (5.8% versus 3.6%) [7]. Symptomatic high-grade block and asystole are far less common.

In this report, we describe the cases of three consecutive patients with ACS who developed serious bradyarrhythmias after ticagrelor, ranging from high-grade atrioventricular (AV) block that responded to withdrawal of the drug to refractory conduction disease that required a permanent pacemaker.

## Case presentation

None of the three patients was receiving a beta-blocker, a calcium-channel blocker, digoxin, ivabradine, or any antiarrhythmic drug at the time of the arrhythmia, and the presenting electrocardiogram in every case showed a normal PR interval of 0.12 seconds and a normal QRS duration of 0.08 seconds with no pre-existing conduction block.

Case 1

A 58-year-old woman, known to be hypothyroid and taking thyroxine 50 micrograms daily, presented with exertional angina of 10 days' duration that was relieved by rest and associated with sweating. She had no breathlessness, palpitations, giddiness, or syncope. Her heart rate was 84 beats per minute and blood pressure 130/80 mmHg. The resting 12-lead electrocardiogram is shown in Figure 1a. Troponin was negative, glycated hemoglobin (HbA1c) was 7.0%, low-density lipoprotein cholesterol was 136 mg/dL, and thyroid-stimulating hormone (TSH) was 3.2 mIU/L. Echocardiography showed hypokinesia of the mid-anteroseptum, apical septum, and left ventricular apex with preserved systolic function (ejection fraction 54%) and grade I diastolic dysfunction. Coronary angiography demonstrated a 95% stenosis of the mid left anterior descending artery (LAD) and a 50% lesion in the second obtuse marginal branch (Figure 1b). She underwent PCI to the proximal-to-mid LAD with a 3.0 by 38 mm everolimus-eluting stent (Figure 1c), and a 180 mg loading dose of ticagrelor was given before the procedure. She was discharged the following day.

**Figure 1:**
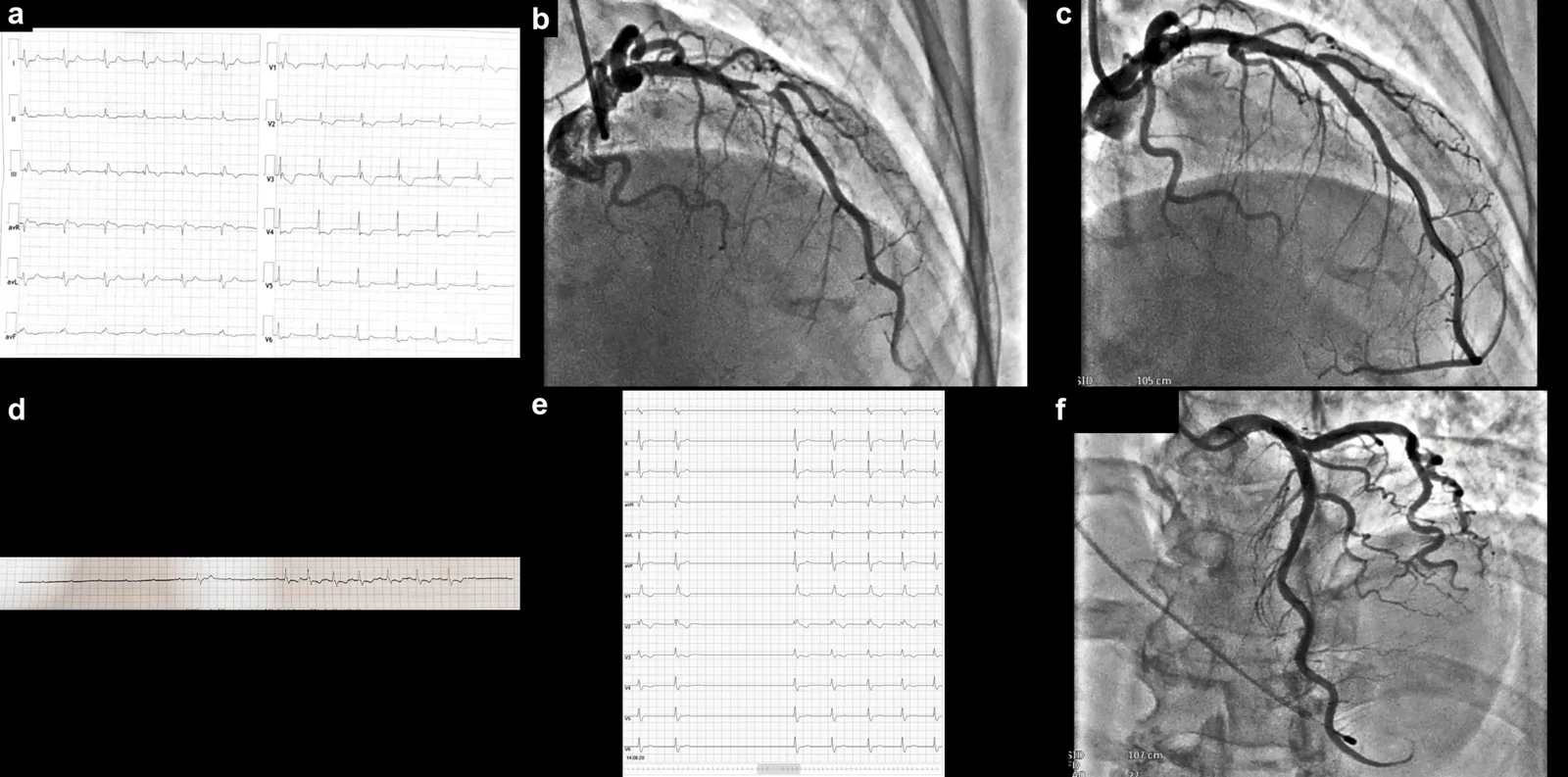
Case 1. (a) 12-lead electrocardiogram. (b) Coronary angiogram showing the mid left anterior descending artery stenosis. (c) Result after drug-eluting stent implantation. (d) Telemetry strip showing a prolonged asystolic pause. (e) High-grade atrioventricular block with a ventricular rate of about 20 beats per minute. (f) Angiogram showing the patent stent with the temporary pacing lead in situ.

The patient was readmitted the next evening with giddiness and a single pre-syncopal episode. There was no angina, breathlessness, or palpitation, no orthostatic hypotension, and no neurological deficit. The heart rate was 74 beats per minute and the blood pressure 120/80 mmHg. The electrocardiogram showed no dynamic ischemic changes and no AV block, and echocardiography confirmed preserved left ventricular function. She was admitted for observation with a 24-hour Holter recording. She had recurrent giddiness and pre-syncope in hospital, during which the heart rate fell to 42 beats per minute. During that episode, telemetry captured a prolonged asystolic pause (Figure 1d), and a 12-lead recording showed high-grade AV block with a ventricular rate of about 20 beats per minute (Figure 1e).

The patient was transferred to the intensive care unit, and an emergency transjugular temporary pacing wire was placed. A check coronary angiogram confirmed a patent LAD stent, with the temporary pacing lead in situ (Figure 1f). Ticagrelor-associated bradyarrhythmia was suspected. The arrhythmia had begun about one hour after re-loading; ticagrelor was stopped, and prasugrel was substituted, and she remained intermittently dependent on the temporary wire for about 48 hours until the effect of ticagrelor had worn off. The wire was removed after an extended Holter recording showed no further sinus pauses, and by three days the rhythm had recovered. At follow-up, she had no further giddiness and no bradycardia.

Case 2

A 48-year-old woman with systemic hypertension, type 2 diabetes mellitus, treated hypothyroidism, dyslipidemia, and focal segmental glomerulosclerosis on medical treatment presented with chest pain. An outside hospital diagnosed an anterior wall myocardial infarction and referred her for primary PCI, giving an 180 mg loading dose of ticagrelor in the emergency department.

On arrival, her heart rate was 102 beats per minute and blood pressure 130/70 mmHg, and the electrocardiogram confirmed an anterior wall myocardial infarction (Figure 2a). TSH was markedly raised at 84.9 mIU/L. Echocardiography showed a dilated left atrium and extensive anterior and septal hypokinesia with moderate left ventricular systolic dysfunction (ejection fraction 38%) and grade II mitral regurgitation (Video 1). Coronary angiography showed a 100% proximal LAD occlusion in a right-dominant circulation (Figure 2b), with non-critical disease of the proximal circumflex and first obtuse marginal. Primary PCI was performed to the ostio-proximal LAD with a 3.0 by 33 mm drug-eluting stent (Figure 2c). She was transferred to the intensive care unit.

**Figure 2:**
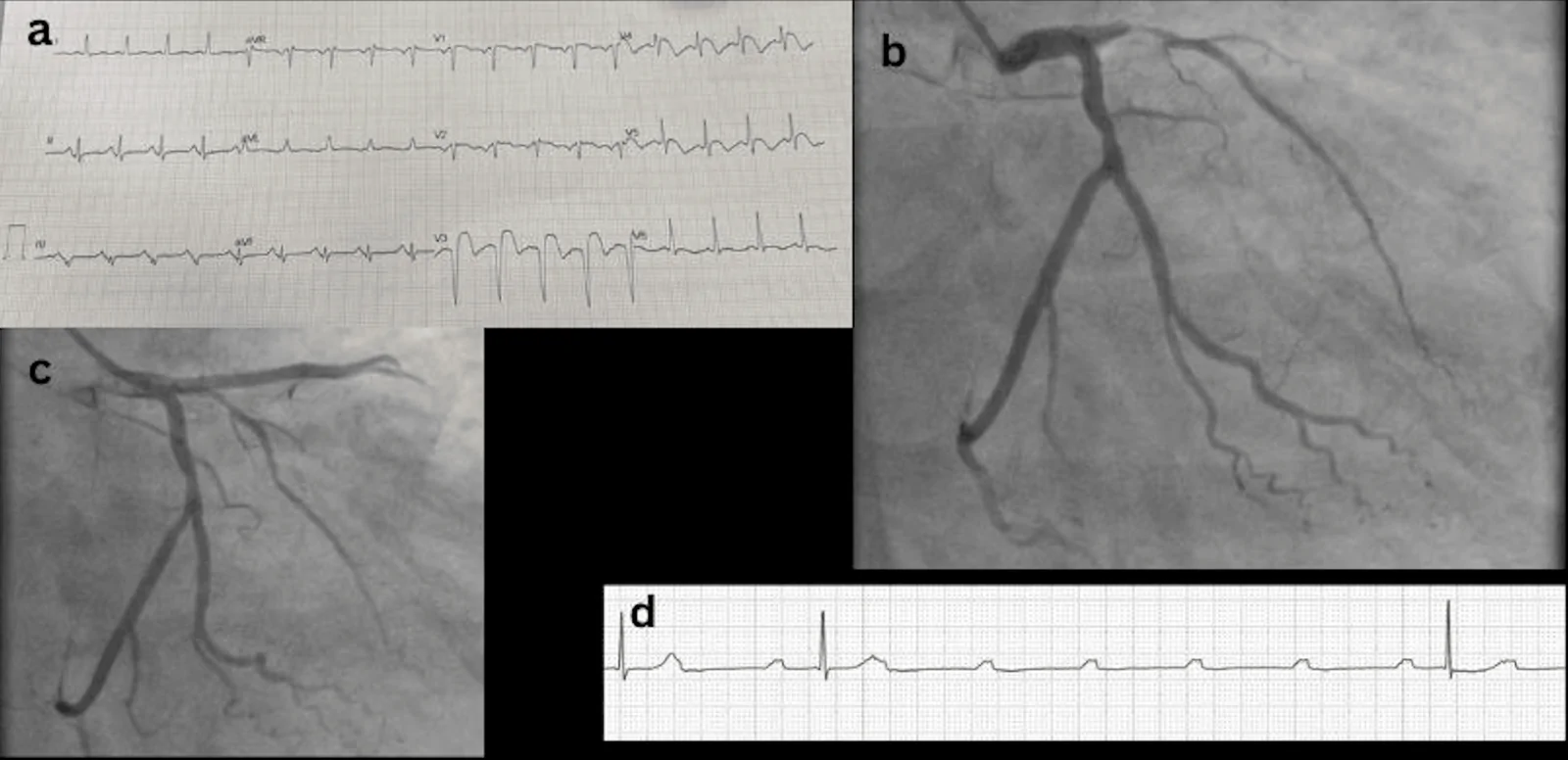
Case 2. (a) 12-lead ECG showing ST elevation in V1- V4 suggestive of anterior wall myocardial infarction. (b) Coronary angiogram showing 100% proximal LAD occlusion. (c) A DES deployed from ostial to proximal LAD (d) Rhythm showing high degree AV block. LAD: left anterior descending artery; DES: drug-eluting stent

**Video 1:** Echocardiography of Case 2 showing a dilated left atrium; anterior and septal hypokinesia with moderate left ventricular systolic dysfunction (ejection fraction 38%) and grade II mitral regurgitation.

About half an hour after loading, the patient suddenly became unresponsive. The monitor showed P waves without conducted QRS complexes, suggestive of high-grade AV block. Cardiopulmonary resuscitation (CPR) was begun. After two doses of intravenous epinephrine, return of spontaneous circulation was achieved, and the electrocardiogram showed sinus rhythm. An angiogram was done, which showed a patent LAD stent with slow flow. Tirofiban and nicorandil infusions were started. The monitor showed high-grade AV block with a ventricular rate below 40 beats per minute (Figure 2d), and an emergency ultrasound-guided temporary pacing wire was placed through the right internal jugular vein. Ticagrelor-associated bradyarrhythmia was suspected, and it was stopped and substituted with prasugrel. The heart rate normalized, and she was dependent on the temporary wire for about six hours. The wire was then kept as a standby, switched off on the third day and removed on the fourth, and by three days the rhythm had recovered. The patient was monitored for a further 24 hours without recurrence and was discharged in stable condition on prasugrel. On follow-up, she was stable with no symptoms or signs on prasugrel therapy.

Case 3

A 77-year-old man with dyslipidemia presented with central chest pain from the early hours that was associated with giddiness but not with breathlessness or palpitations. His heart rate was 88 beats per minute and blood pressure 140/80 mmHg. The electrocardiogram showed sinus rhythm with ST-segment elevation in leads II, III, aVF, and V5 to V6, and ST-segment depression in V1, V2, aVR, and aVL, suggestive of an acute inferoposterior myocardial infarction (Figure 3a). Echocardiography showed inferolateral hypokinesia with mild left ventricular dysfunction, with an ejection fraction of 53% (Video 2). Thyroid function was normal (TSH, 1.4 mIU/L). He was loaded with ticagrelor and aspirin in the emergency room. Coronary angiography showed a 100% acute total occlusion of the proximal right coronary artery (RCA) in a right-dominant circulation (Figure 3b), and primary PCI was performed from the proximal to mid RCA with a 3.5 by 44 mm drug-eluting stent (Figure 3c), after which he was observed in the intensive care unit.

**Figure 3:**
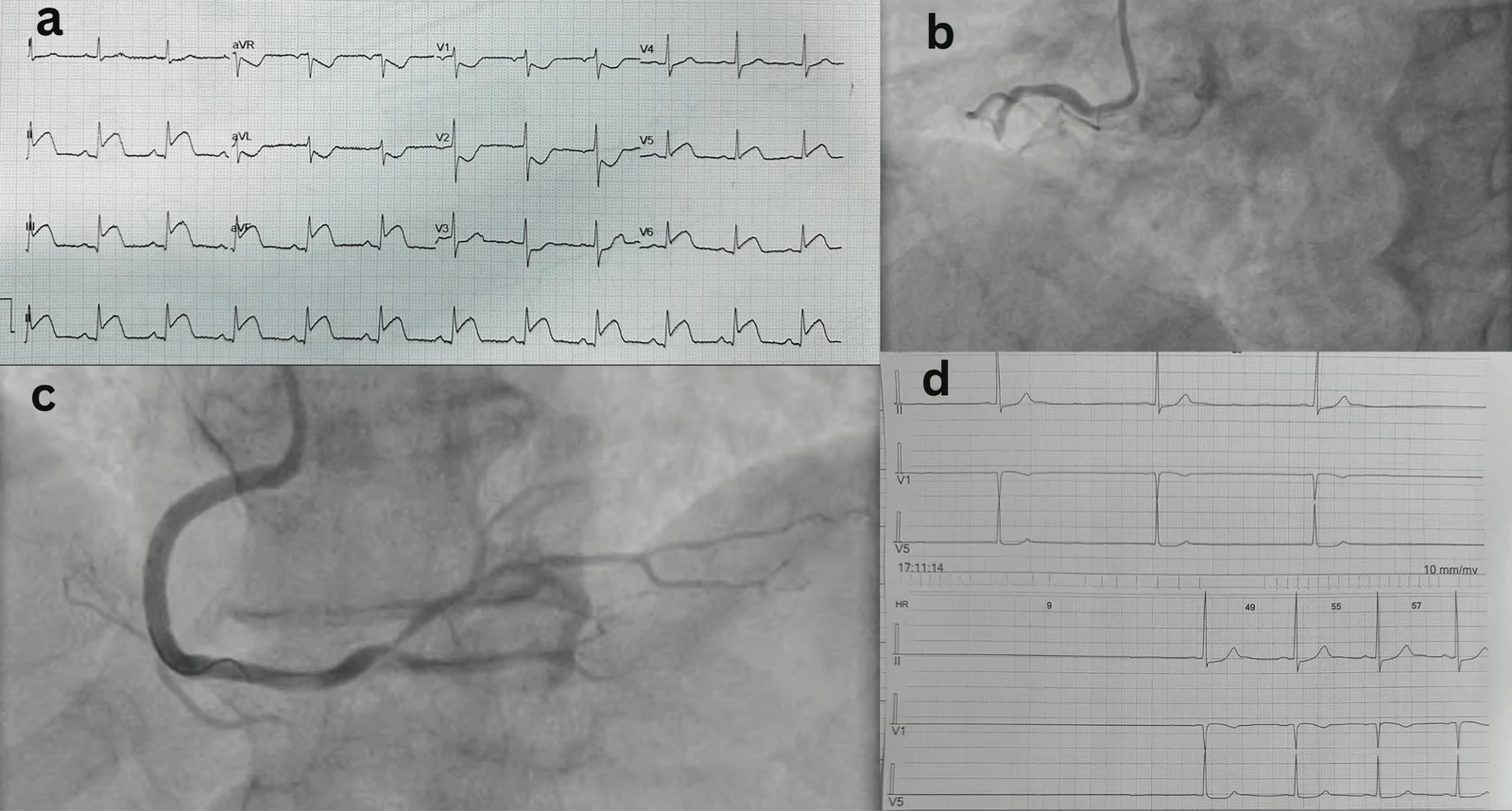
Cases 3. (a) 12 -lead electrocardiogram showing an ST segment elevation in Lead II,III, aVF, V5 to V6 and ST segment depressions in I, aVL, V1,V2,V3; suggestive of inferior-posterior wall myocardial infarction. (b) Coronary angiogram showing proximal RCA - 100% occluded. (c) Primary PCI from proximal to mid RCA using DES (d) Post-procedure Holter monitoring showing sinus pauses RCA: right coronary artery; DES: drug-eluting stent

**Video 2:** Echocardiogram in Case 3 showing inferolateral hypokinesia with mild left ventricular dysfunction (ejection fraction 53%)

On the first day after the procedure, the patient developed intermittent AV block followed by runs of non-sustained ventricular tachycardia, and then became suddenly unresponsive with a 10-second period of asystole and ventricular fibrillation, the heart rate falling to a nadir of nine beats per minute. He was defibrillated (a total of 200 joules) and received two cycles of CPR, intravenous magnesium sulfate, and lidocaine before return of spontaneous circulation. An angiogram was done, which showed a patent stent in the RCA, and a temporary pacing wire was placed. The asystole and bradycardia were attributed to ticagrelor, and the drug was switched to prasugrel. He required pacing intermittently for two days. The temporary wire was kept in place while the effect of ticagrelor washed out, and when it was switched off after three days, he developed a further sinus pause of 4.2 seconds that required one cycle of CPR, recorded on Holter (Figure 3d). The temporary pacing wire was re-inserted. Because the conduction disturbance persisted despite withdrawal of ticagrelor, a dual-chamber permanent pacemaker was implanted. His subsequent course was uneventful, and he was discharged in stable condition on prasugrel. At follow-up, he remains intermittently dependent on his pacemaker.

The clinical characteristics of the three patients are summarized in Table 1, the temporal course in Table 2, and the Naranjo causality assessment in Table 3.

**Table 1:** Clinical characteristics, arrhythmia, and outcome of the three patients. ACS: acute coronary syndrome; AV: atrioventricular; CPR: cardiopulmonary resuscitation; DES: drug-eluting stent; FSGS: focal segmental glomerulosclerosis; LAD: left anterior descending artery; NSTEMI: non-ST-elevation myocardial infarction; NSVT: non-sustained ventricular tachycardia; PCI: percutaneous coronary intervention; RCA: right coronary artery; RV: right ventricular; STEMI: ST-elevation myocardial infarction; VF: ventricular fibrillation

Parameter	Case 1	Case 2	Case 3
Age (years), sex	58, female	48, female	77, male
Relevant history	Hypothyroidism (treated)	Hypertension, diabetes, hypothyroidism, dyslipidemia, FSGS	Dyslipidemia
ACS type	NSTEMI	Anterior STEMI	Inferoposterior and RV STEMI
Culprit vessel	Mid LAD (95%)	Proximal LAD (100%)	Proximal RCA (100%)
PCI / stent	DES 3.0 x 38 mm to LAD	DES 3.0 x 33 mm to LAD	DES 3.5 x 44 mm to RCA
Ejection fraction	54%	38%	53%
Thyroid-stimulating hormone	3.2 mIU/L	84.9 mIU/L	1.4 mIU/L
Concomitant AV-nodal blocking drug	None	None	None
Baseline PR interval	0.12 seconds	0.12 seconds	0.12 seconds
Baseline QRS duration	0.08 seconds	0.08 seconds	0.08 seconds
Pre-existing conduction block	None	None	None
Ticagrelor exposure	180 mg load before PCI	180 mg load in emergency department	180 mg loaded before PCI
Bradyarrhythmia	High-grade AV block, asystolic pause	High-grade AV block, cardiac arrest	AV block, asystole, NSVT and VF
Nadir heart rate	42/minute	<40/minute	9/minute
Cardiac arrest / CPR	No	Yes (epinephrine x2)	Yes (200 J, magnesium, lidocaine)
Temporary pacing	Yes (about 48 hours)	Yes (removed day 4)	Yes (two episodes)
Antiplatelet change	Ticagrelor stopped, prasugrel started	Ticagrelor stopped, prasugrel started	Ticagrelor stopped, prasugrel started
Definitive outcome	Recovered, no pacemaker	Recovered, no pacemaker	Permanent dual-chamber pacemaker
Follow-up	No further bradycardia	No further symptoms	Intermittent pacemaker dependence

**Table 2:** Temporal course from ticagrelor loading to arrhythmia, pacing, and recovery Intervals are approximate and are measured from the ticagrelor loading dose

Case	Loading to arrhythmia onset	Temporary-pacing dependence	Rhythm recovery or washout	Definitive outcome
Case 1	About 1 hour	About 48 hours	Recovered by about 3 days	No pacemaker
Case 2	About 0.5 hour	About 6 hours	Recovered by about 3 days	No pacemaker
Case 3	About 0.5 hour	Intermittent over about 3 days (two episodes)	Did not recover; 4.2 seconds sinus pause after 3-day washout	Permanent pacemaker

**Table 3:** Naranjo adverse drug reaction probability assessment for each case The Naranjo scale grades an adverse drug reaction as definite (9 or more), probable (5 to 8), possible (1 to 4), or doubtful (0 or less) [8]. A competing ischemic cause (item 5) and the absence of a rechallenge (item 4) limited the score in each case.

Naranjo item	Case 1	Case 2	Case 3
1. Previous conclusive reports on this reaction	+1	+1	+1
2. Adverse event appeared after the drug was given	+2	+2	+2
3. Improved when the drug was withdrawn	+1	+1	0
4. Reappeared when the drug was readministered	0	0	0
5. Alternative causes could have caused the reaction	-1	-1	-1
6. Reappeared with placebo	0	0	0
7. Drug detected in toxic concentration	0	0	0
8. Dose-response relationship	0	0	0
9. Previous similar reaction on prior exposure	0	0	0
10. Event confirmed by objective evidence	+1	+1	+1
Total score	4	4	3
Category	Possible	Possible	Possible

## Discussion

Ticagrelor in ACS and the recognition of bradyarrhythmia

Ticagrelor is a cornerstone of antiplatelet therapy after ACS. In the PLATO trial, which enrolled 18,624 patients, it reduced the composite of vascular death, myocardial infarction, and stroke compared with clopidogrel without an increase in overall major bleeding [1]. Current guidelines do not list bradyarrhythmia among the routine reasons to avoid ticagrelor [3], which is one reason these events are easily overlooked. In this series, all three patients developed serious bradyarrhythmias after ticagrelor was given for ACS. All three required temporary pacing, two had a cardiac arrest with successful resuscitation, and one required a permanent pacemaker.

Contributory factors and the differential diagnosis

Several factors other than ticagrelor can slow the heart after ACS, and we considered each. First, ongoing ischemia of the conduction system is the principal alternative, because atrioventricular block is more frequent in inferior myocardial infarction, where the right coronary artery supplies the atrioventricular node, than in other territories [9]. In every patient, however, the culprit lesion had been treated successfully, and the stent was patent on a repeat angiogram, which made ongoing ischemia from stent failure an unlikely sole explanation. Second, no patient was receiving a beta-blocker, a calcium-channel blocker, digoxin, or an antiarrhythmic drug, so a pharmacological co-factor other than ticagrelor was excluded. Third, the events tended to occur at rest and at night, when vagal tone is highest. Fourth, two of the three patients had hypothyroidism. In Case 1, the patient was biochemically controlled (TSH 3.2 mIU/L), and in Case 3, the patient was euthyroid, but the markedly raised TSH of 84.9 mIU/L in Case 2 reflects severe hypothyroidism, which is itself a recognized cause of sinus bradycardia and impaired conduction and may have lowered the threshold for the observed block [10]. The abrupt onset within hours of loading, the absence of pre-existing conduction disease on the baseline electrocardiogram, and the recovery after drug withdrawal in Cases 1 and 2 nonetheless point to ticagrelor as an important contributor acting on this susceptible background.

Proposed mechanism

Ticagrelor binds reversibly to the P2Y12 receptor at a site distinct from the adenosine diphosphate binding pocket. Beyond platelet inhibition, it inhibits the equilibrative nucleoside transporter 1, reducing cellular re-uptake of adenosine and raising its extracellular concentration [4]. Ticagrelor inhibits adenosine uptake by human erythrocytes and potentiates adenosine-mediated coronary hyperemia in animal models [11,12]. Adenosine has powerful negative chronotropic and dromotropic effects, slowing the sinoatrial node and depressing AV nodal conduction through A1 receptors, and in ACS extracellular adenosine is already increased by ischemic release [13]. By further reducing adenosine re-uptake, ticagrelor is thought to amplify these effects and to produce sinus bradycardia, sinus pauses, and AV block. When this pharmacological effect is superimposed on ischemic injury to the conduction system, as in inferior or right ventricular infarction, the two mechanisms are likely to act together, which is the most plausible explanation for the severity seen in our patients. Pharmacovigilance data support a genuine signal, since an analysis of the United States Food and Drug Administration (FDA) Adverse Event Reporting System found disproportionate reporting of sinus arrest, complete AV block, and bradycardia with ticagrelor [14].

Causality assessment

To move beyond a temporal association, we applied the Naranjo adverse drug reaction probability scale to each case [8]. The reaction followed drug administration, is supported by previous reports [15-20] and objective electrocardiographic documentation, and improved after withdrawal in Cases 1 and 2, while the presence of ACS as an alternative cause and the absence of a rechallenge limited the score. Each case reached a total consistent with a possible adverse drug reaction (Table 3). We therefore present ticagrelor as a likely contributor rather than the proven sole cause, in keeping with the design limitations of a case series.

Interpretation of case 3

In Case 3, the patient required permanent dual-chamber pacing despite discontinuation of ticagrelor, and three explanations merit consideration. The bradyarrhythmia could represent a direct and unusually severe adverse drug reaction, the unmasking of latent conduction-system disease in a 77-year-old man, or irreversible ischemic injury to the conduction system from the inferoposterior and right ventricular infarction. The temporal link to loading and the initial asystole favor a ticagrelor contribution, but the persistence of a 4.2-second sinus pause after a full three-day washout, in the territory most associated with conduction-system infarction, indicates that irreversible ischemic injury was also present. We interpret Case 3 as a multifactorial event in which ticagrelor and ischemic conduction-system injury acted together, which is why withdrawal of the drug alone did not restore conduction.

Comparison with literature

Ticagrelor-associated bradyarrhythmia has previously been reported almost entirely as isolated cases, summarized in Table 4. Reported events, typically appearing within hours of a 180 mg loading dose and resolving after a switch to prasugrel or clopidogrel, include an 11-second sinus pause [15], an 18.5-second ventricular pause [16], cyclical sinus bradycardia with transient complete AV block and pauses of up to five seconds [17], a 12-second sinus pause [18] and a 45-second asystolic pause [19]. Against this background, our series is notable in three respects. The time to onset was short in every case, from about half an hour to about one hour after loading, which is at the earlier end of the reported range. Two patients presented at the peri-arrest end of the severity spectrum rather than with isolated pauses. A permanent pacemaker had previously been required only in a single elective case in which asystole of up to 10 seconds was provoked by intra-procedural adenosine [20], whereas our third patient required permanent pacing after spontaneous ACS, most likely because of superimposed ischemic conduction-system injury.

**Table 4:** Reported cases of ticagrelor-associated bradyarrhythmia compared with the present series. *This case involved an elective (non-ACS) procedure in which asystole was provoked by intraprocedural adenosine and is included as a mechanistic comparator. ACS: acute coronary syndrome; AV: atrioventricular; CAD: coronary artery disease; CHB: complete heart block; CPR: cardiopulmonary resuscitation; FFR: fractional flow reserve; HR: heart rate; IV: intravenous; LAD: left anterior descending artery; LCx: left circumflex artery; NSTEMI: non-ST-elevation myocardial infarction; PCI: percutaneous coronary intervention; PPM: permanent pacemaker; RCA: right coronary artery; RV: right ventricle; STEMI: ST-elevation myocardial infarction; VF: ventricular fibrillation; VT: ventricular tachycardia.

Study (author, year)	Age / sex	Setting	Time to event	Arrhythmia	Lowest rate / longest pause	Management	PPM	Outcome
Present report, Case 1	58 / F	NSTEMI, LAD PCI	About 1 hour after loading	High-grade AV block, pause	Rate about 20/minute	Stop ticagrelor, prasugrel, temporary pacing	No	Recovered
Present report, Case 2	48 / F	Anterior STEMI, LAD PCI	About 0.5 hours after loading	Complete heart block, cardiac arrest	Rate below 40/minute	Stop ticagrelor, prasugrel, epinephrine, temporary pacing	No	Recovered
Present report, Case 3	77 / M	Inferoposterior and RV STEMI, RCA PCI	About 0.5 hours after loading	AV block, asystole, VT/VF	9/min; pauses 10 seconds, then 4.2 seconds	Stop ticagrelor, prasugrel, CPR, defibrillation, temporary pacing	Yes	Recovered, pacemaker-dependent
Kotaru (2021) [15]	58 / M	NSTEMI, LCx PCI	About 2 hours after 180 mg load	Sinus bradycardia, asystolic pause, syncope	11-second pause	Switch to prasugrel	No	Recovered
Low (2019) [16]	59 / F	NSTE-ACS, LCx PCI	3 hours after 180 mg load	Ventricular pauses	18.5-second pause	Switch to clopidogrel, temporary wire	No	Recovered
Waldmann (2018) [17]	75 / F	ACS, PCI	Hours after PCI	Cyclical sinus bradycardia, transient CHB	Below 30/minute; 5-second pauses	Switch to clopidogrel	No	Recovered
Pavlatos (2023) [18]	58 / M	Inferior STEMI, RCA PCI	About 38 hours	Sinus pauses, junctional escape	12-second pause	Switch to clopidogrel	No	Recovered
Gorzynska-Schulz (2025) [19]	67 / F; 67 / M	NSTEMI; anterior STEMI	Day 4; first night	CHB with asystole; sinus pauses	45-second pause	IV theophylline, switch to prasugrel	No	Recovered
Al-Bayati (2021) [20]*	64 / F	Elective, stable CAD, adenosine FFR	Intra-procedure	Sinus arrest/asystole	Up to 10-second asystole	Switch to clopidogrel, aminophylline	Yes	Recovered

Seriousness and severity of the adverse drug reaction

These were serious adverse drug reactions. Each event was life-threatening and required urgent intervention; all three patients needed temporary pacing: two sustained a cardiac arrest with successful resuscitation, and one required a permanent pacemaker, although none was fatal. In severity, the reactions lay at the extreme end of the bradyarrhythmia spectrum, with high-grade AV block, a heart-rate nadir of nine beats per minute, and asystolic pauses of up to 10 seconds. This degree of severity, arising within hours of a loading dose, distinguishes the present series from the isolated and largely self-limiting pauses that dominate the published reports (Table 4) and underlines why prompt recognition and rhythm support are essential. The high seriousness of the reaction should be weighed against the balanced Naranjo causality, which reached only the possible category in each case because ACS provided a competing cause.

Clinical implications and management

Certain patients appear more susceptible, including older patients, those with inferior or right ventricular infarction affecting the blood supply to the conduction system, those with pre-existing conduction disease or sinus node dysfunction, and those taking other AV nodal blocking drugs. Identification rests on a high index of suspicion and rhythm monitoring, with early 12-lead electrocardiography when a patient reports giddiness, pre-syncope or syncope and ambulatory Holter recording for intermittent symptoms. Management is graded to severity, as set out in Figure 4.

**Figure 4:**
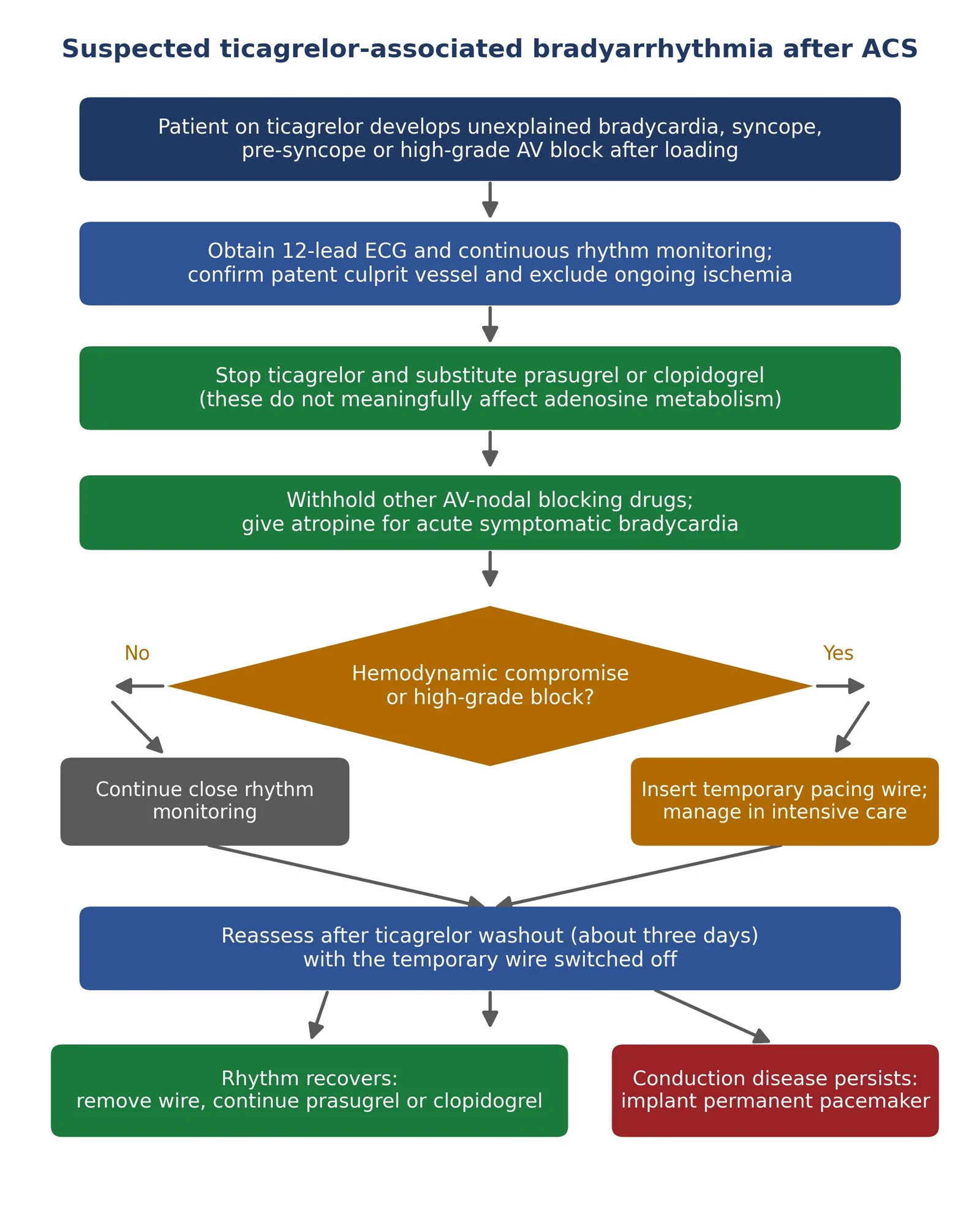
Stepwise management algorithm for suspected ticagrelor-associated bradyarrhythmia after acute coronary syndrome ACS, acute coronary syndrome; AV, atrioventricular; ECG, electrocardiogram.

Asymptomatic pauses can be monitored. When the bradyarrhythmia is symptomatic, ticagrelor should be stopped and replaced with prasugrel or clopidogrel, which do not meaningfully affect adenosine metabolism. Other AV nodal blocking drugs should be withheld; atropine can be used for acute symptomatic bradycardia, and a temporary pacing wire should be placed when there is hemodynamic compromise or high-grade block. A permanent pacemaker is reserved for the minority in whom conduction disease persists after drug washout, as in our third patient.

Limitations

This is a series of three patients from a single center and cannot establish incidence or prove causation. The Naranjo score reached only the possible category in each case, chiefly because ACS provides a competing explanation and a rechallenge was neither feasible nor ethical. Serum ticagrelor concentrations and adenosine levels were not measured. The strengths are the consistent temporal pattern, absence of AV nodal blocking co-medication, documented recovery on withdrawal in two patients, and objective electrocardiographic and angiographic characterization of each case.

## Conclusions

Ticagrelor can contribute to serious bradyarrhythmias in patients with ACS, including high-grade AV block, prolonged pauses, and cardiac arrest, and the diagnosis is easily missed because the drug is given routinely and events can occur after the culprit lesion has been treated. Clinicians should suspect ticagrelor adverse effects in any patient with unexplained bradycardia or syncope after its administration, monitor the rhythm early, and substitute with prasugrel or clopidogrel when symptoms occur. Bradyarrhythmia usually resolves once ticagrelor is withdrawn, but as this series shows, it can be severe enough to cause cardiac arrest, and permanent pacing may be required when the drug effect is superimposed on ischemic injury to the conduction system rather than resolving with drug washout.
